# Plant Disease Classification: A Comparative Evaluation of Convolutional Neural Networks and Deep Learning Optimizers

**DOI:** 10.3390/plants9101319

**Published:** 2020-10-06

**Authors:** Muhammad Hammad Saleem, Johan Potgieter, Khalid Mahmood Arif

**Affiliations:** 1 Department of Mechanical and Electrical Engineering, School of Food and Advanced Technology, Massey University, Auckland 0632, New Zealand; H.Saleem@massey.ac.nz; 2 Massey Agritech Partnership Research Centre, School of Food and Advanced Technology, Massey University, Palmerston North 4442, New Zealand; J.Potgieter@massey.ac.nz

**Keywords:** plant disease classification, convolutional neural network, deep learning, validation accuracy, F1-score

## Abstract

Recently, plant disease classification has been done by various state-of-the-art deep learning (DL) architectures on the publicly available/author generated datasets. This research proposed the deep learning-based comparative evaluation for the classification of plant disease in two steps. Firstly, the best convolutional neural network (CNN) was obtained by conducting a comparative analysis among well-known CNN architectures along with modified and cascaded/hybrid versions of some of the DL models proposed in the recent researches. Secondly, the performance of the best-obtained model was attempted to improve by training through various deep learning optimizers. The comparison between various CNNs was based on performance metrics such as validation accuracy/loss, F1-score, and the required number of epochs. All the selected DL architectures were trained in the PlantVillage dataset which contains 26 different diseases belonging to 14 respective plant species. Keras with TensorFlow backend was used to train deep learning architectures. It is concluded that the Xception architecture trained with the Adam optimizer attained the highest validation accuracy and F1-score of 99.81% and 0.9978 respectively which is comparatively better than the previous approaches and it proves the novelty of the work. Therefore, the method proposed in this research can be applied to other agricultural applications for transparent detection and classification purposes.

## 1. Introduction

In order to match the food demand, agricultural problems should be addressed by advanced techniques. In this regard, the agricultural industries are focusing on artificial intelligence methods. Several traditional machine learning (ML) algorithms have been used to perform various agricultural operations. On top of that, deep learning (DL) produced significant developments in the agricultural field of research. This is due to the automatic feature extraction capability of the deep learning algorithms. Among several agricultural problems, the successful classification of plant diseases is vital to improve the quality/quantity of agricultural products and reduce an undesirable application of chemical sprayers such as fungicide/herbicide. Therefore, it is an emerging research topic to advance agricultural automation. This agricultural task has a complexity due to the resemblance in the occurrence of the plant containing diseases. In this regard, several studies have been conducted to improve the classification of plant disease. 

Many conventional machine learning (ML) models have been applied for plant disease classification [1,2]. Similarly, advanced imaging techniques including hyperspectral [3,4,5,6,7] and multispectral imaging [8] have also been used for plant/leaf disease identification. However, after the evolution of deep learning (DL), many state-of-the-art architectures, including AlexNet [9,10,11,12,13,14], Visual Geometry Group (VGG) [10,11,13,15,16], DenseNet [16], Inception-v4 [16] and ResNet [11,13,14,16], got promising results for the classification of plant disease. In this regard, several studies proved the significance of deep learning-based methods as compared to the traditional ML techniques. For example, a well-known DL model named GoogLeNet outperformed the ML algorithms including Support Vector Machine (SVM) and Random Forest (RF) models for the classification of disease in tomato leaves [9]. Another research showed the effectiveness of Convolutional Neural Networks (CNN) in comparison with the other state-of-the-art techniques such as Radial Basis Function Neural Network (RBFNN), Particle Swarm Optimization (PSO), and SVM for the classification of defects in mango leaves [17]. An article proposed a CNN model to identify diseases in apple leaves, which provided higher accuracy than SVM, Back-Propagation Neural Network model (BPNN), AlexNet, GoogLeNet, ResNet, and VGG models [18]. In [19], a CNN model was proposed to classify the disease in the leaves of PlantVillage dataset; its performance was better than the ML techniques such as SVM, Decision Tree (DT), Logistics Regression (LR), and K-Nearest Neighbor (KNN) models. This model also performed better than the well-known DL architectures including AlexNet, ResNet, VGG-16, and Inception-v3. Therefore, this article focuses on the DL-based models for the classification of plant disease.

Different approaches have been adopted to enhance the results of plant disease classification including modified versions of well-known DL models, various training techniques, data augmentation techniques, cascaded versions of two successful DL architectures, etc. [6]. For example, the famous GoogLeNet model was improved to achieve better testing accuracy for the identification of maize leaf disease in a small period due to its lesser number of parameters [20]. Similarly, inspired by the AlexNet model, a modified CNN architecture was proposed that had a lesser number of filters in convolutional layers and number of nodes, which apparently reduced overall parameters as compared to the original model and successfully identified the disease in tea leaves [21]. By using an extended version of the PlantVillage dataset, two modified versions of MobileNet models were proposed and their performance was compared with the original model (MobileNet), AlexNet, and VGG models [22]. Another research proposed a cascaded version of DL architecture to classify disease in apple leaves and it achieved better results as compared to AlexNet, GoogLeNet, VGG-16, Inception-v3, and various versions of ResNet models [23]. Moreover, several visualization techniques were also utilized along with DL models to highlight the disease spots in several plant species [9,24,25,26]. Few studies have been conducted to further advance the research of plant disease classification by using various training techniques. In [12], the performance of two well-known DL models (AlexNet and GoogLeNet) was compared, which were trained from transfer learning and scratch techniques. Reference [16] implemented ResNet, VGG, Inception-v4, and DenseNet models by using a fine-tuning technique. Another research compared the performance of DL architectures including AlexNet, ResNet, DenseNet, SqueezeNet, Inception-v3, and VGG by training through transfer learning and scratch techniques [24].

The research in deep learning has been progressing with the passage of time by introducing various methods to achieve remarkable outcomes. For example, in [27], a random search method was proposed for tuning the hyperparameters of the neural network to reduce forecasting errors. Similarly, various recent studies proposed the optimizations algorithms to find the optimal value of hyperparameters of DL architectures [28]. Moreover, deep learning requires an optimization algorithm to update the weight parameters and reduce the losses. Therefore, various deep learning optimizers have been developed by the research community to achieve better results in image classification tasks. These optimizers produce a significant improvement in the performance of DL models. In the context of plant disease classification, the previous researches either focused on the modification of state-of-the-art DL models or the deployment of various training techniques. However, none of the previous studies has proposed an improvement in the plant disease classification by state-of-the-art DL optimizers through a comparative study. In this regard, this article presents a comprehensive comparative analysis to perform plant disease classification in two steps. In the first step, the performance of 18 convolutional neural networks was evaluated: 10 famous/well-known DL architectures that were previously used for several image recognition tasks, six recently published modified versions that were derived from the famous DL models, and two cascaded/hybrid versions that were developed from two efficient DL algorithms; the second step was applied to improve the performance of the best-obtained model by training with various deep learning optimizers including RMSProp, Adam, Adadelta, Adamax, and Adagrad. For a comprehensive evaluation, validation accuracy/loss, F1-score, and the number of epochs (required to converge training and validation plots) were compared. The PlantVillage dataset was selected for this research, which contains disease in 14 different plant species. The successful/better classification results obtained in a large variety of dataset classes confirm that the method presented in this article can also be applied to other datasets related to plant disease. Furthermore, the better results obtained by this research will be useful for future studies regarding the real-time classification and detection of plant disease in a single framework. Moreover, the proposed methodology could also be adopted to other agricultural applications.

The rest of the paper is organized as follows: Section 2 presents the details of the dataset, hardware/software specifications, DL architectures, DL optimizers, and specifications required to train the DL models. Section 3 presents the results to indicate the performance of all the well-known, modified, and cascaded/hybrid versions of DL models along with the improvement in the performance of best-obtained models by using various deep learning optimizers, and finally, Section 4 describes the concluding remarks along with some future recommendations.

## 2. Materials and Methods

**Figure 1:**
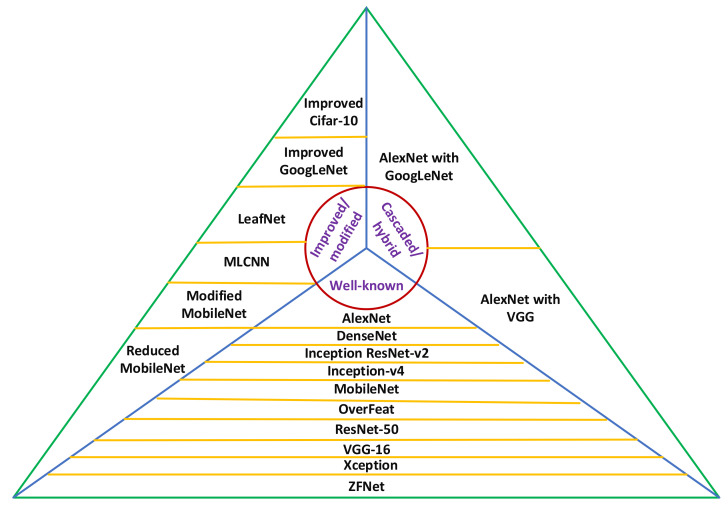
Three categories of DL architectures: well-known, improved/modified, and cascaded/hybrid versions. MLCNN: Multi-label Convolutional Neural Network, VGG: Visual Geometry Group.

**Figure 2:**
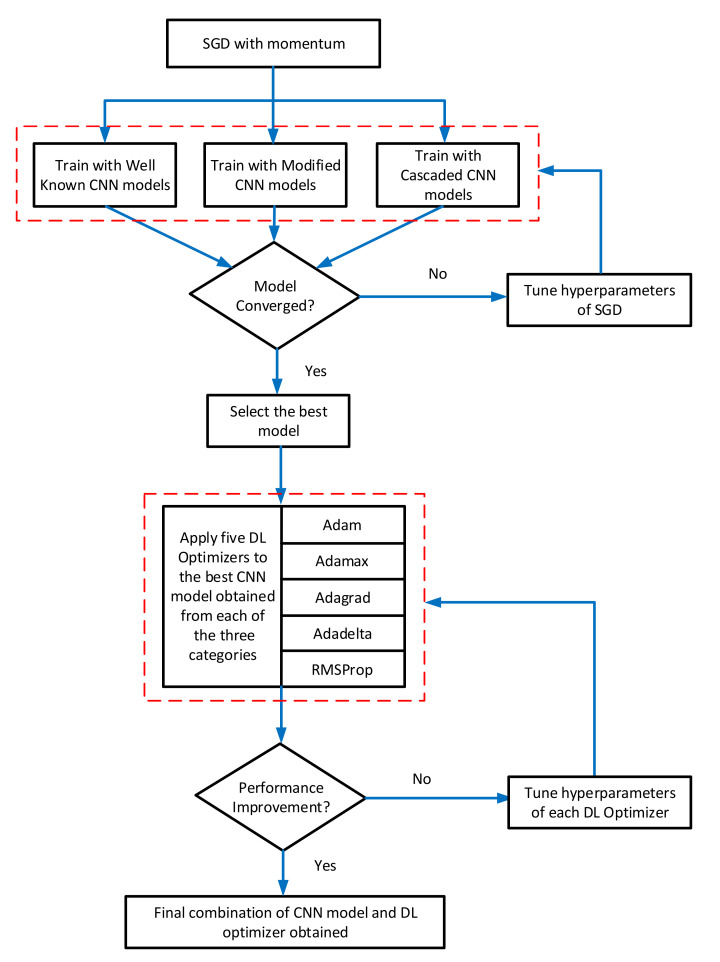
The methodology of this research. CNN: Convolutional Neural Network, SGD: Stochastic Gradient Descent.

The Convolutional Neural Networks (CNNs) are mostly used for image classification tasks. Therefore, in this research, the performance of many state-of-the-art CNN architectures was evaluated for the classification of plant diseases. The modified and cascaded versions of DL architectures were also considered, which were recently published in prominent research articles related to plant disease classification. Figure 1 shows all the 18 DL architectures considered for this research. These models were divided into three categories: well-known, modified/improved, and cascaded/hybrid versions. An overall methodology of this research is presented in Figure 2. Firstly, the Stochastic Gradient Descent (SGD) with momentum optimizer was selected to train the CNN models due to its fast convergence ability [24]. Then, 18 CNN architectures were trained on the PlantVillage dataset and their convergence to the final training/validation values was observed to update the hyperparameters. Next, the CNN models were compared in terms of training and validation accuracy/loss, and F1-score. This led us to apply the DL optimization algorithms for further improvement in the performance of those CNN architectures, which achieved the highest F1-score in their particular category. The novelty of the work is proved by getting the most suitable combination of the CNN model and DL optimizer, which provided considerably better result as compared to the previous researches.

### 2.1. Dataset

**Figure 3:**
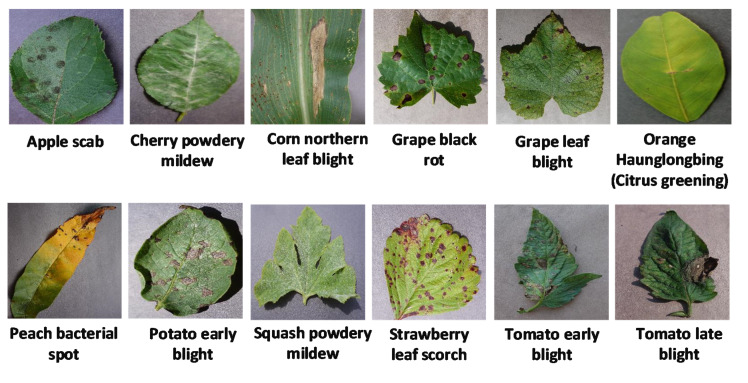
Some of the plant diseases from the PlantVillage dataset [29].

All the DL models were trained on a publicly available dataset called PlantVillage [29], which contains a total of 54,306 images containing 38 different healthy/diseased leaves related to their 14 plant species (some of the plant diseases are shown in Figure 3). The size of the images was changed to 224 × 224 × 3 and normalization was considered by dividing the values of pixel by 255 for making it suitable for the initial values of the models. The dataset was divided by 70%, 20%, and 10% into three categories to avoid overfitting: training, validation, and testing datasets, respectively [22].

### 2.2. Software and Hardware Specifications

The DL architectures were programmed in Python language due to the availability of very useful libraries and DL frameworks. Keras with TensorFlow backend was utilized to build the architectures. CuDNN library was installed as it increases the speed of training and works with TensorFlow. All the experiments were carried out on a Graphical Processing Unit (NVIDIA Quadro K2200) having the specifications: 4GB memory, 640 CUDA cores, 1045 MHz core clock, and 80 GB/sec memory bandwidth.

### 2.3. Deep Learning Architectures

After the development of the AlexNet architecture, a revolutionary period of state-of-the-art CNN architectures was started for many image classification tasks. Therefore, in this article, we considered very popular and successful CNN models such as AlexNet [30], OverFeat [31], VGG-16 [32], ZFNet [33], ResNet-50 [34], Inception ResNet-v2 [35], Inception-v4 [35], MobileNet [36], DenseNet-121 [37] and Xception [38].

Some researchers proposed improved/modified versions of state-of-the-art DL architectures to achieve better/more results for classifying the diseases of plant species. Among them, we have considered improved GoogLeNet [20], inspired by the famous GoogLeNet model [39], Cifar-10 [20], LeafNet [23], a multilayer convolutional neural network (MLCNN) [17] derived from the AlexNet model [30], and modified and reduced MobileNet [22] inspired by the MobileNet model [36]. Some cascaded/hybrid versions of DL architectures have also been considered in this article such as a cascaded form of the well-known AlexNet with GoogLeNet models as described in [18] and a hybrid DL architecture of AlexNet with VGG models (AgroAVNET) as proposed in [40].

### 2.4. Deep Learning Optimizers

The Stochastic Gradient Descent (SGD) was used to train all the DL models during the first step of the proposed method. After getting the best DL architecture, an improvement in the classification of plant disease was also attempted. In this regard, we used five state-of-the-art deep learning optimizers to train those DL models which attained the highest validation accuracy and F1-score in the first step of the analysis. Few characteristics of these optimizers are provided as under:

- SGD: This is one of the simplest deep learning optimizers. A static learning rate for all the parameters requires in the duration of whole training and it has a fast convergence ability [41].
- Adagrad: This optimizer uses different learning rates for every parameter in the model. It updates the learning rate according to the frequency of the update of each parameter [42].
- RMSProp: To reduce the training time observed in Adagrad, the RMSProp optimizing functions were proposed and its learning rate decays exponentially [43].
- Adadelta: This is an extended version of Adagrad optimizer and accumulates the previous gradients over a fixed time window which ultimately ensures the continuation of learning even after many iterations. Adadelta used Hessian approximation to ensure the update direction in the negative gradient and eliminated the learning rate from update rule [44].
- Adam: The Adaptive moment estimation method (Adam) evaluates adaptive learning rates from the first and second moments of gradients for various parameters [45]. It has combined advantages of two extended versions of the SGD method that are Adagrad and RMSProp. In contrast with the RMSProp, it calculates the average of the second moment of gradient and it also utilizes the previous gradients to speed up learning [45].
- Adamax: A different version of Adam was also proposed in [45] which is based on the infinity norm and could be useful for sparse parameter updates like word embeddings.

### 2.5. Training Specifications

All the DL models were trained from scratch on the PlantVillage dataset. The hyperparameters were tuned by the random search method [46]. The internal covariate shift problem occurs on the neural network because of the variation in the distribution of input data due to a change in the number of parameters in the previous layer. This problem was addressed by Batch Normalization which is a very useful technique for a high learning rate [47]. For training all the DL models, the ReLU activation function was used as it is computationally efficient [24,30] and reduces the possibility of the gradient vanishing. The specifications of all the DL optimizers are summarized in Table 1.

## 3. Results and Discussion

**Figure 4:**
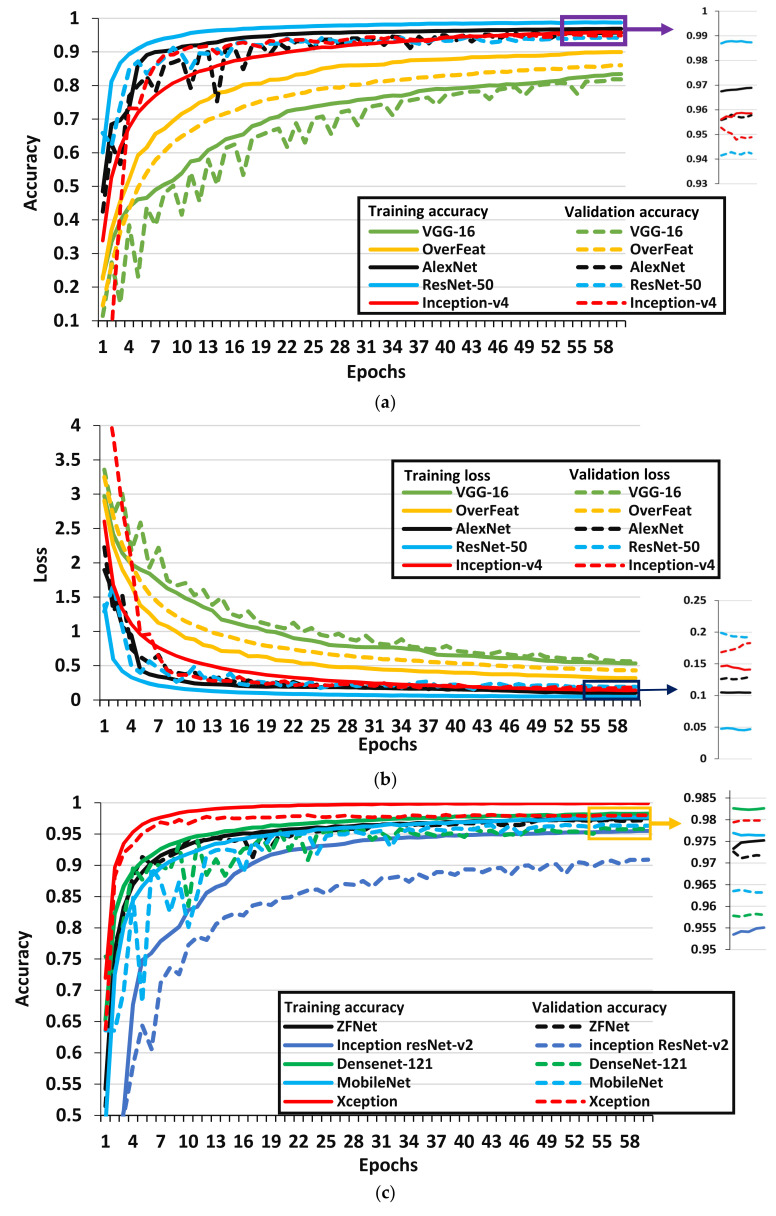

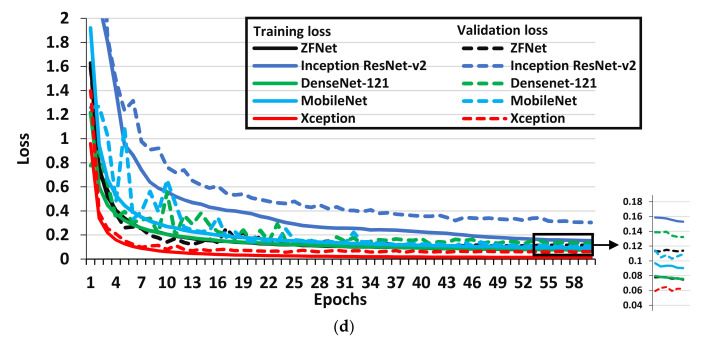
Performance plots of well-known CNN architectures. (**a**), (**b**) provide training and validation accuracy/loss of VGG-16, OverFeat, AlexNet, ResNet-50 and Inception-v4 architectures. (**c**), (**d**) provide training and validation accuracy/loss of ZFNet, Inception ResNet-v2, DenseNet-121, MobileNet and Xception architectures.

**Figure 5:**
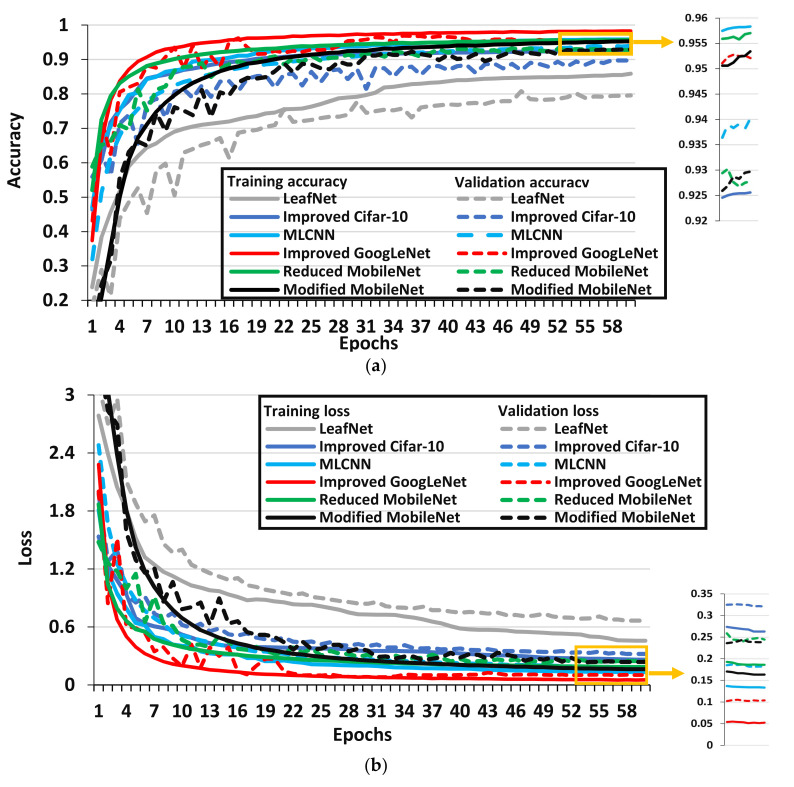
Performance plots of modified versions of CNN architectures. (**a**) Provides training and validation accuracy, (**b**) provides training and validation loss.

**Figure 6:**
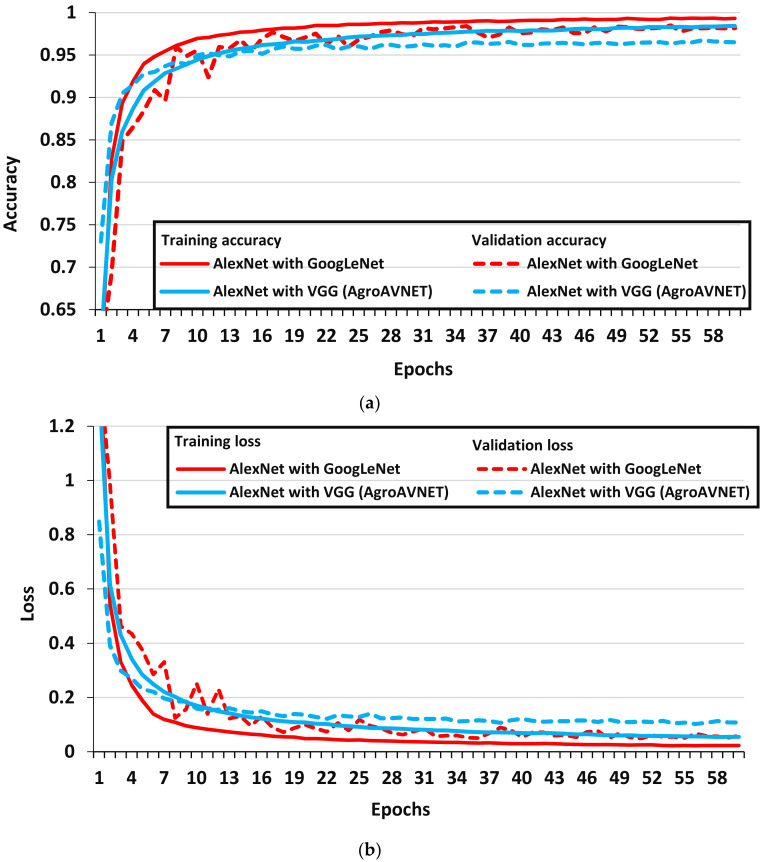
Performance plots of cascaded/hybrid versions of CNN architectures. (**a**) Provides training and validation accuracy, (**b**) provides training and validation loss.

This section first presents the comparative analysis of DL architectures to select the best model which leads to the results obtained regarding the improvement in the performance of the best-suited models by using various DL optimization algorithms. All the results were evaluated in terms of training, validation accuracy/loss, and F1-score. The F1-score is considered an important performance metric especially for the case when there is an uneven distribution in the classes just such as the PlantVillage dataset (for example, the Potato healthy class contains the least number of images (152), whereas, the Citrus greening has the highest number of images (5507) [29]). Therefore, the model/optimizer that attained the highest F1-score was considered the most suitable architecture for the classification of plant disease. The performances of all DL architectures are represented by line graphs (Figure 4, Figure 5 and Figure 6), and it was empirically observed that they required 60 epochs (an epoch is a complete cycle of training on each image sample in the training dataset) at which training/validation accuracy and loss were converged. The overall performance of DL architectures is also summarized in Table 2.

### 3.1. Step-1: Comparative Analysis of Deep Learning Architectures 

#### 3.1.1. Performance of Well-Known CNN Architectures

The performance of well-known CNN architectures is presented in Figure 4, and it indicates that there is no sign of underfitting (the problem occurs during the training of deep learning models according to which the model does not train accurately if training loss does not change or it continuously decreases) and overfitting (the problem at which the model does not perform appropriately for new data/validation dataset or validation loss decreases to some extent then suddenly increases for the remaining epochs). Overall, 10 well-known CNN architectures were considered. A few important observations from Figure 4 and Table 2 were made:

- The Xception model attained the highest validation accuracy, F1-score, and lowest validation loss among all the well-known CNN models. Therefore, this model can be undoubtedly considered as the best CNN architecture to classify plant disease on the PlantVillage dataset. It implies that the concept of a modified version of depth-wise separable convolution [38] in the Xception model is a useful way to obtain higher classification results. Moreover, this DL model converged to its final value at the 34^th^ epoch which is the least number of epochs as compare to all the other DL architectures. On the other hand, it required a significant amount of time to complete one epoch (around 3400 sec). Therefore, future studies should propose another version of DL architecture that can achieve Xception-level accuracy and require smaller training time for each epoch.
- The second highest F1-score/validation accuracy was attained by ZFNet architecture. Hence, a smaller filter size and the increased number of activation maps used in ZFNet architectures (as compared to AlexNet) improved its performance.
- Then, MobileNet, DenseNet, and AlexNet architectures have also achieved a good F1-score followed by Inception-v4, ResNet-50, and Inception ResNet-v2 architectures. The MobileNet is a comparatively more preferable model due to its lower number of parameters which reduced its computation time significantly. The depthwise and pointwise convolutional layers helped to achieve a better classification result. Therefore, a CNN model could be proposed in future research based on the MobileNet architecture. Moreover, this model required a lower number of epochs to achieve its final accuracy and loss as compare to DenseNet and AlexNet models (as shown in Table 2).
- From Table 2, it is also noticed that the DL models, such as Inception-v4, Inception ResNet-v2, OverFeat, and VGG-16, required 58-59 number of epochs to converge training/validation plots (also shown in Figure 4), which significantly increased their training time.
- The VGG-16 and OverFeat were found unsuitable models for plant disease classification as they achieved lower validation accuracy/F1-score and higher validation loss as compared to the other well-known DL architectures. The smaller filter size of the VGG model degraded its performance. However, the larger filter size of the OverFeat model significantly reduced its training time but they were not enough to provide a noticeable classification performance. Additionally, they had a higher number of parameters (in millions) which slow down their training time effectively.

#### 3.1.2. Performance of Modified CNN Architectures

In this article, six modified/improved versions of CNN architectures were also considered. Their performance is presented in Figure 5 from which the following points are discussed:

- The improved GoogLeNet architecture achieved the best performance in terms of validation accuracy/loss and F1-score among all the modified versions of CNN architectures by utilizing the concept of the Inception module from the original GoogLeNet model. Moreover, it got the final value of accuracy and loss in 53 epochs which is the least as compared to other modified/improved versions of the DL models considered in this article, but it required more training time to complete one epoch as compared to the models like Modified and Reduced MobileNet.
- The MLCNN architecture provided a good F1-score due to the inclusion of a dropout layer after each max pooling layer and a reduction in the number of filters of the starting convolution layers in the original AlexNet architecture. However, due to a higher number of parameters, this modified DL architecture required considerably higher training time per epoch.
- The two versions of MobileNet named Modified and Reduced MobileNet models achieved an acceptable F1-score closed to each other. These modified versions of DL architecture used depthwise separable convolutional layers, which helped to attain a good classification result, and they had six times fewer parameters than the original MobileNet model which reduced their training time per epoch.
- Moreover, there were some models like Improved Cifar-10 and LeafNet models that had a lower number of parameters which increased their speed of training per epoch. The Improved Cifar-10 model achieved a noticeable F1-score, but the reduced parameters of the LeafNet model were not enough to obtain a good F1-score/validation accuracy. Therefore, it is not a suitable model to classify diseases in the selected dataset. It is also observed that these two models required a higher number of epochs as compare to other modified versions of DL architectures. Hence, future research could comprise of proposing a DL model such as Improved Cifar-10 and LeafNet for reducing the training time, but some convolutional layers should be added to attain acceptable validation/testing accuracy.

#### 3.1.3. Performance of Cascaded/Hybrid CNN Architectures

Figure 6 presents the performance of cascaded/hybrid version of CNN models as explained below:

- The cascaded AlexNet with GoogLeNet architecture outperformed all the DL models in terms of validation accuracy; moreover, except for the Xception architecture, this model achieved the highest F1-score among all the DL architectures considered in this research (as shown in Table 2). Although it required almost 57 epochs to reach its final accuracy/loss values (as shown in Figure 6), but it completed one epoch in a smaller period, which clearly shows its effectiveness in terms of training time. There were a few important modifications in the original AlexNet model, which helped to extract the features of plants containing disease including smaller convolution kernel in different layers, the inclusion of max-pooling layer, cascading the Inception module with the modified AlexNet layers, and convolutional layers after Inception to replace two fully connected layers [18].
- Moreover, a hybrid version of AlexNet with VGG architectures has also been studied, and it provided good performance in terms of validation accuracy (as shown in Figure 6) and F1-score, but it had the highest number of parameters which significantly increased its training time to complete each epoch. This model performed well due to the utilization of concepts such as normalization and selection of filter depth from AlexNet and VGG models, respectively [40].

### 3.2. Step-2: Improvement in Classification Results by Deep Learning Optimizers

In this article, an improvement in the performance of CNN architectures has also been attempted by training the best models (obtained from the previous step) through different deep learning optimization functions. In this regard, the best DL model was selected from each of the three categories such as the Xception, Improved GoogLeNet, and cascaded version of AlexNet with GoogLeNet models. Table 3 summarizes the results obtained by using various optimization algorithms. Some important observations can be made as follows:

- Considerable changes were observed in training/validation accuracy, loss, precision, recall, and F1-score by training the DL models through various deep learning optimizers.
- Adam and Adadelta were the most successful optimizers for all the three selected DL architectures.
- The Xception model trained with the Adam optimizer achieved the highest validation accuracy and F1-score of 99.81% and 0.9978, respectively, which clearly show the effectiveness of the proposed approach. Moreover, these results are better than previous studies that used the same dataset but different approaches [12,16,19,24]. Therefore, the methodology proposed in this article could be used for various other agricultural operations.
- The cascaded AlexNet with GoogLeNet and improved GoogLeNet models achieved their best classification results by using the Adadelta and Adam optimizers, respectively.
- However, a degradation in the performance has also been observed when optimizing functions were changed from SGD to Adagrad and RMSProp for Xception and cascaded models, respectively.
- It is also noticed that the Improved GoogLeNet showed its lowest validation accuracy/F1-score when it was trained by the SGD optimizer.

## 4. Conclusions and Future Recommendations

In this article, a comprehensive comparative analysis has been performed between various state-of-the-art deep learning architectures divided into three categories namely well-known, modified, and cascaded versions. Moreover, the performance of the best-obtained models was further improved by using various deep learning optimization algorithms. It was found that the Xception, Improved GoogLeNet and cascaded version of AlexNet with GoogLeNet models obtained the highest validation accuracy and F1-score in their respective category. When these three DL models were trained by using various deep learning optimizers, the Xception model trained by the Adam optimizer achieved the highest F1-score of 0.9978 which suggests that this combination of the CNN model and the optimization algorithm is the most suitable way to classify the plant disease. This research provided us some interesting future directions for upcoming research given as follows:

- Various deep learning optimizers such as Adam, and Adadelta, can also be used to enhance research on other agricultural applications, such as crop/weed discrimination, classification of weeds, plant recognition, etc.
- The classification performance of the other datasets related to plant disease could also be improved by adopting the methodology proposed in this research.
- Furthermore, although the Xception model provided the best results according to the analysis provided in this article, it required a significant amount of time to complete each epoch. Therefore, an attempt should be made to achieve an Xception level accuracy with small training time.

## Figures and Tables

**Table 1:** Hyperparameters of the deep learning optimizers.

Optimizers	Specifications
**SGD**	learning rate = 0.001, weight decay = 0.0005, momentum = 0.9, nesterov = False
**Adagrad**	learning rate = 0.001, epsilon = 1 × 10^−7^
**RMSProp**	learning rate = 0.001, rho = 0.9, epsilon = 1 × 10^−7^
**Adadelta**	learning rate = 1.0, rho=0.95, epsilon= 1 × 10^−6^
**Adam**	learning rate = 0.001, beta1 = 0.9, beta2 = 0.999, epsilon = 1 × 10^−8^, amsgrad = False
**Adamax**	learning rate = 0.002, beta1 = 0.9, beta2 = 0.999, epsilon = 1 × 10^−8^

**Table 2:** Training and validation accuracy/loss, precision, recall, and F1-score along with the number of parameters, training time, and epochs required to train deep learning architectures (in the order of the lowest to the highest F1-score).

Deep Learning Architectures	Parameters (in Millions)	Epochs Required to Train the Model	Training Time (in Hours)	Training Accuracy	Validation Accuracy	Training Loss	Validation Loss	Precision	Recall	F1-score
**LeafNet**	0.324 M	59	5.95	0.8590	0.7961	0.4563	0.6658	0.7946	0.7971	0.7958
**VGG-16**	138 M	59	38.13	0.8339	0.8189	0.5328	0.5651	0.8182	0.8194	0.8188
**OverFeat**	141.8 M	58	6.75	0.8995	0.8603	0.3201	0.4330	0.8592	0.8628	0.8610
**Improved Cifar-10**	2.43 M	58	6.08	0.9256	0.8974	0.2628	0.3205	0.8944	0.8960	0.8952
**Inception ResNet v2**	54.3 M	58	32.83	0.9551	0.9091	0.1530	0.3047	0.9075	0.9105	0.9089
**Reduced MobileNet**	0.5 M	55	11.72	0.9570	0.9278	0.1860	0.2442	0.9269	0.9267	0.9268
**Modified MobileNet**	0.5 M	53	6.38	0.9534	0.9297	0.1632	0.2385	0.9278	0.9265	0.9271
**ResNet-50**	23.6 M	55	26.33	0.9873	0.9423	0.0468	0.1923	0.9351	0.9358	0.9354
**MLCNN**	78 M	57	67.33	0.9583	0.9402	0.1335	0.1820	0.9386	0.9411	0.9398
**Inception v4**	41.2 M	59	52.92	0.9586	0.9489	0.1410	0.1828	0.9410	0.9466	0.9438
**Improved GoogLeNet**	6.8 M	53	9.67	0.9829	0.9521	0.0522	0.1038	0.9528	0.9539	0.9533
**AlexNet**	60 M	54	6.10	0.9689	0.9578	0.1046	0.1298	0.9563	0.9570	0.9566
**DenseNet-121**	7.1 M	56	28.75	0.9826	0.9580	0.0758	0.1323	0.9581	0.9569	0.9575
**MobileNet**	3.2 M	47	14.70	0.9764	0.9632	0.0903	0.1090	0.9624	0.9612	0.9618
**Hybrid AlexNet with VGG (AgroAVNET)**	238 M	54	49.90	0.9841	0.9649	0.0546	0.1078	0.9626	0.9674	0.9650
**ZFNet**	58.5 M	47	6.47	0.9752	0.9717	0.0746	0.1139	0.9746	0.9751	0.9748
**Cascaded AlexNet and GoogLeNet**	5.6 M	57	6.5	0.9931	0.9818	0.0229	0.0592	0.9749	0.9751	0.9750
**Xception**	22.8 M	34	56.28	0.9990	0.9798	0.0140	0.0621	0.9764	0.9767	0.9765

**Table 3:** Performance of deep learning optimizers applied to train cascaded AlexNet with GoogLeNet, Improved GoogLeNet, and Xception models.

Optimizers	Training Accuracy	Validation Accuracy	Training Loss	Validation Loss	Precision	Recall	F1-score
**Cascaded AlexNet with GoogLeNet**							
**SGD**	0.9931	0.9818	0.0229	0.0592	0.9749	0.9751	0.9750
**RMSProp**	0.9894	0.9757	0.0482	0.1479	0.9746	0.9613	0.9679
**Adagrad**	0.9956	0.9824	0.0153	0.0547	0.9815	0.9782	0.9798
**Adamax**	0.9990	0.9859	0.0029	0.0574	0.9828	0.9795	0.9811
**Adam**	0.9989	0.9857	0.0039	0.0750	0.9836	0.9836	0.9836
**Adadelta**	0.9993	0.9873	0.0024	0.0696	0.9846	0.9856	0.9851
**Improved GoogLeNet**							
**SGD**	0.9829	0.9521	0.0522	0.1038	0.9528	0.9539	0.9533
**RMSProp**	0.9723	0.9685	0.1780	0.2272	0.9692	0.9666	0.9679
**Adagrad**	0.9889	0.9718	0.0350	0.0930	0.9651	0.9618	0.9634
**Adamax**	0.9998	0.9847	8.782 × 10^−4^	0.0875	0.9792	0.9826	0.9809
**Adam**	0.9992	0.9904	0.0026	0.0434	0.9859	0.9872	0.9864
**Adadelta**	0.9991	0.9905	0.0022	0.0567	0.9828	0.9879	0.9861
**Xception**							
**SGD**	0.9990	0.9798	0.0140	0.0621	0.9764	0.9767	0.9765
**RMSProp**	0.9998	0.9924	6.922 × 10^−4^	0.0433	0.9877	0.9920	0.9900
**Adagrad**	0.9987	0.9621	0.0164	0.1460	0.9682	0.9505	0.9593
**Adamax**	1.0000	0.9889	0.0012	0.0415	0.9902	0.9874	0.9888
**Adam**	1.0000	0.9981	6.890 × 10^−4^	0.0178	0.9981	0.9975	0.9978
**Adadelta**	1.0000	0.9906	8.407 × 10^−4^	0.0364	0.9926	0.9887	0.9906
